# Developing Capacities of Community Health Workers in Sexual and Reproductive, Maternal, Newborn, Child, and Adolescent Health: A Mapping and Review of Training Resources

**DOI:** 10.1371/journal.pone.0094948

**Published:** 2014-04-15

**Authors:** Nguyen Toan Tran, Anayda Portela, Luc de Bernis, Kristen Beek

**Affiliations:** 1 School of Public Health and Community Medicine, University of New South Wales, Sydney, Australia; 2 Department of Maternal, Newborn, Child and Adolescent Health (MCA), World Health Organization, Geneva, Switzerland; 3 United Nations Population Fund (UNFPA), Geneva Office, Geneva, Switzerland; 4 Faculty of Health, University of Technology, Sydney, Australia; School of Population Health, The University of Queensland, Australia

## Abstract

**Background:**

Given country demands for support in the training of community health workers (CHWs) to accelerate progress towards reaching the Millennium Development Goals in sexual and reproductive health and maternal, newborn, child, and adolescent health (SR/MNCAH), the United Nations Health Agencies conducted a synthesis of existing training resource packages for CHWs in different components of SR/MNCAH to identify gaps and opportunities and inform efforts to harmonize approaches to developing the capacity of CHWs.

**Methods:**

A mapping of training resource packages for CHWs was undertaken with documents retrieved online and from key informants. Materials were classified by health themes and analysed using agreed parameters. Ways forward were informed by a subsequent expert consultation.

**Results:**

We identified 31 relevant packages. They covered different components of the SR/MNCAH continuum in varying breadth (integrated packages) and depth (focused packages), including family planning, antenatal and childbirth care (mainly postpartum haemorrhage), newborn care, and childhood care, and HIV. There is no or limited coverage of interventions related to safe abortion, adolescent health, and gender-based violence. There is no training package addressing the range of evidence-based interventions that can be delivered by CHWs as per World Health Organization guidance. Gaps include weakness in the assessment of competencies of trainees, in supportive supervision, and in impact assessment of packages. Many packages represent individual programme efforts rather than national programme materials, which could reflect weak integration into national health systems.

**Conclusions:**

There is a wealth of training packages on SR/MNCAH for CHWs which reflects interest in strengthening the capacity of CHWs. This offers an opportunity for governments and partners to mount a synergistic response to address the gaps and ensure an evidence-based comprehensive package of interventions to be delivered by CHWs. Packages with defined competencies and methods for assessing competencies and supervision are considered best practices but remain a gap.

## Introduction

In countries where Millennium Development Goals (MDGs) 4, 5 and 6 are most lagging, there is a need to accelerate progress to improve the health of women and children and address the unmet needs for sexual and reproductive health, and maternal, newborn, child, and adolescent health (SR/MNCAH) of the larger community [1]. This requires investment in and strengthening of national health systems, especially in underserved and hard-to-reach areas [2], [3]. The crisis in human resources for health is one of the most critical factors underlying the poor performance of health systems in resource-constrained settings [4], [5]. This crisis hinders the equitably-distributed delivery of effective interventions for SR/MNCAH [6] and other priority health issues–an estimated 4.3 million health workers are needed to fill the gap in 57 countries in Africa and Asia [7].

Community health workers (CHWs) have been described as lay people who live in the communities where they work and function as a critical link between these communities and the primary health care system [8]. For decades, they have been recognized as playing an important role in connecting women, families, and communities to health services, and in contributing to improvements in the health of mothers, newborns and children [9]–[13]. CHWs can be providers of health promotion and preventive care and also increasingly of curative care, thanks to new rapid diagnostic tests, simplified treatment protocols, and mobile health technologies and support systems [14]. As such, a CHW is defined by WHO as *any health worker who performs functions related to health-care delivery; was trained in some way in the context of the intervention; but has received no formal professional or paraprofessional or tertiary education, should be members of the communities where they work, be selected by the communities, be answerable to the communities for their activities and should be supported by the health system* [15]. The broad application and divergence of this definition at country level as to who is a CHW and what tasks they undertake is noted. It is recognized that several countries have a formal cadre of health workers who do not fall into this of CHW, such as Health Extension Workers in Ethiopia, or “community health workers” in Papua New Guinea, as they receive longer and more comprehensive training and can perform higher-level tasks.

Government institutions, United Nations agencies, and global partners have been repositioning the role that CHWs can play in increasing access to essential quality health services [16]–[19]. However, CHWs are not the answer to weak health systems. Numerous CHW programmes have failed in the past because of unrealistic expectations, poor planning and underestimation of resources required to make these programmes work [20]. Other CHW programmes have been designed to address very narrow and specific needs, often without taking into account the potential of CHWs in SR/MNCAH, and probably to meet the demands of specific projects and partner interests. Often the role of the CHWs is not well-defined and the supporting policies and strategies are not promoted. Due to these factors, CHW programmes have suffered from poor integration into the broader health system. In conjunction with the work of other health cadres, and when deployed within the context of an appropriately financed and organized primary health care system, CHWs can contribute to improving the coverage of essential SR/MNCAH interventions [8], [21].

Over the preceding decade, some countries, UN agencies and partners had embarked on designing and implementing training packages for CHWs for various SR/MNCAH topics, such as home- and community-based newborn and child care [22]–[24]. However recently, in response to the growing body of evidence on the potential role and effectiveness of CHWs, the World Health Organization (WHO) and partners have published global recommendations on task-shifting and evidence-based interventions that CHWs can undertake in the different areas of SR/MNCAH [25]–[28].

Before embarking on the elaboration of additional CHW training curricula on SR/MNCAH as requested by countries, UN agencies and partners felt it was necessary to take stock of existing training tools. A few CHW training resource packages had been independently assessed within their respective projects, but there has not been a global mapping and review of existing training resources packages for CHWs on SR/MNCAH. This paper aims to identify, organize and provide a synthesis of existing training resource materials for CHWs in different components of SR/MNCAH and to determine gaps and opportunities and inform efforts to avoid duplication and harmonize approaches to training CHWs in SR/MNCAH.

## Materials and Methods

### Search Strategy

**Figure 1:**
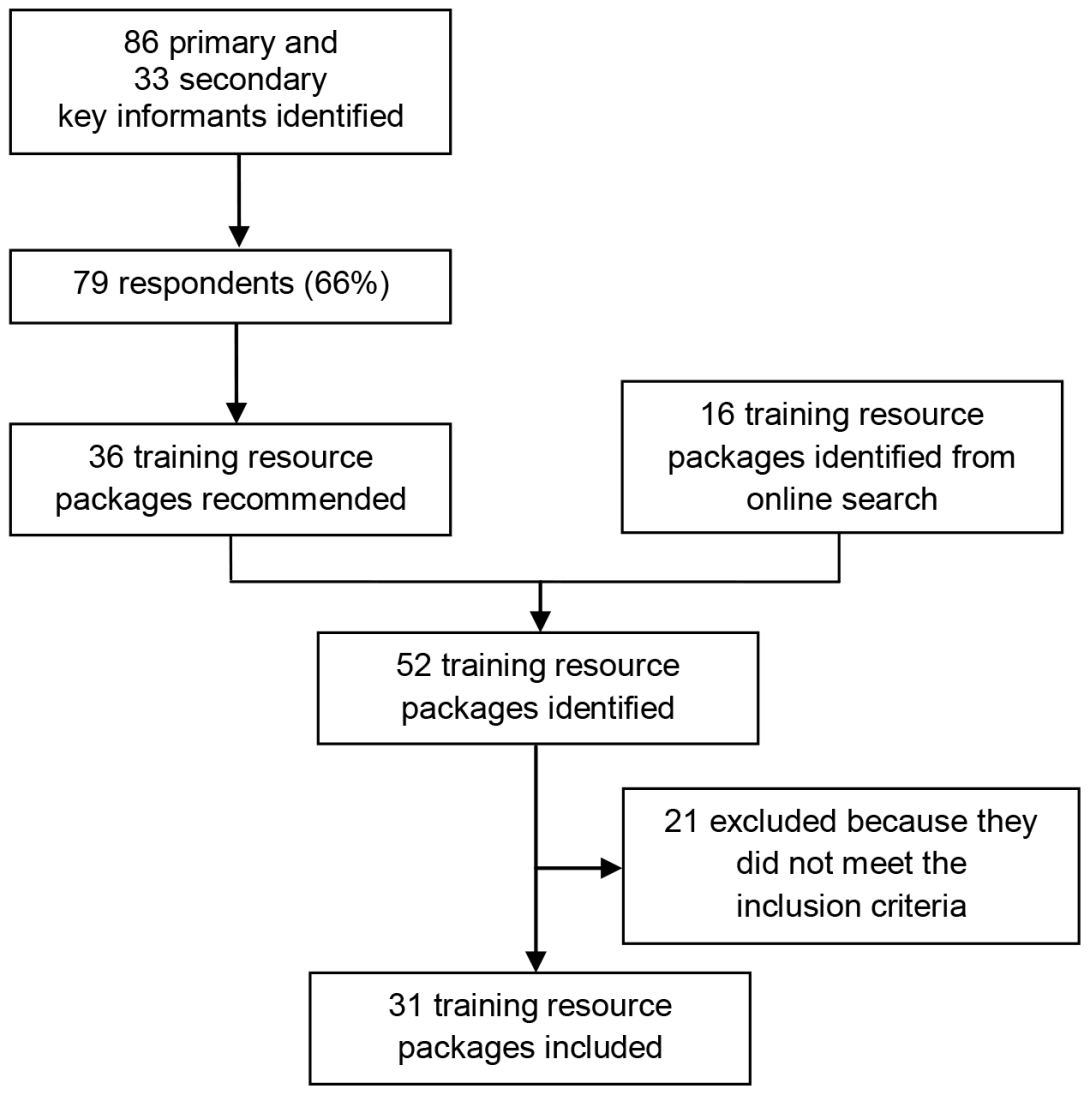
Search strategy.

From October to December 2012, training resource packages were sourced using two different approaches. In the first search strategy, primary key informants from United Nations agencies, NGOs, academic institutions and other partners were contacted by email. They were informed about the objective of the mapping and asked to share training resource materials for CHWs on SR/MNCAH and snowball this request to secondary key informants. Semi-structured interviews were conducted face-to-face or via teleconference with key informants when required to clarify information. An online search was also conducted through targeted (POPLINE, MEDLINE) and general (Google, GoogleScholar) search engines (see Figure 1).

### Inclusion and Exclusion Criteria

Training resource packages targeting CHWs and including both job aids and a training guide on topics related to SR/MNCAH, including HIV, adolescent SRH and gender-based violence, were retained. The following were not included in the analysis: job aid alone, policy guidance alone, programme manager guide alone, materials on community-based activities not related to CHW training. Relevant materials in English, French, and Spanish produced in or after 2000 were included.

### Quality Review, Data Abstraction and Synthesis

**Figure 2:**
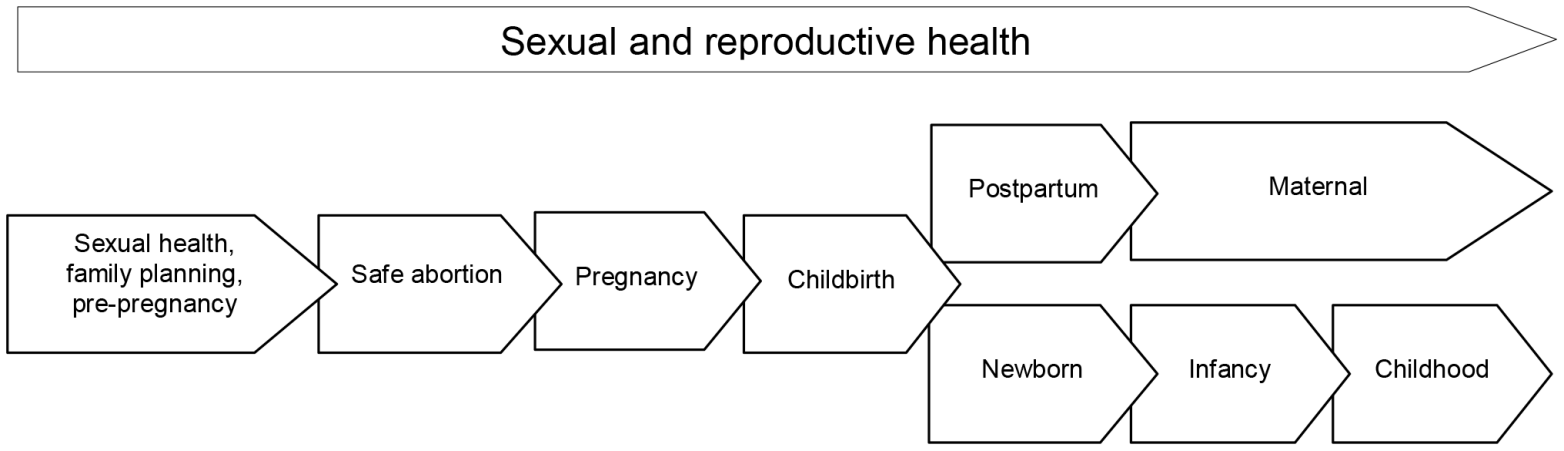
Continuum of sexual and reproductive health and maternal, newborn, child, and adolescent health.

The materials were classified according to the SR/MNCAH continuum of care as defined by WHO [25] (see Figure 2). Information was extracted from the selected training packages as per the following areas: purpose, description, scope and training methodology of the tool; integration of the tool in countries and health systems; strengths and limitations. To assess the strengths and limitations of the tools, the following questions were applied: 1) Did the interventions of the training package match the home/community interventions recommended by WHO? [25]; and 2) Did the training package use: role- and context-specific contents, competency- and practice-based training, supportive supervision, continuing and refresher training, sound training and pedagogy including pre-training, training and post-training steps, inclusion of job aids, and integration of acquired skills into the overall CHW role? Two reviewers carried out the quality review, data abstraction and synthesis of materials and cross-checked their findings. When feasible, the results were verified with the authors or relevant authoring agencies.

### Expert Review

The results of the mapping exercise were presented during a technical consultation on CHWs and SR/MNCAH [29] organized by WHO and the United Nations Population Fund (UNFPA) on behalf of the UN H4+ (the United Nations health-related agencies in SR/MNCAH: UNAIDS, UNFPA, UNICEF, UN Women, WHO, and the World Bank). The meeting was held in Geneva in February 2013 and brought together 32 participants from nine countries, including technical experts and programme managers from UN agencies, academic institutions, and government and non-governmental organizations. The reflections, discussions and consensus from the consultation inform the discussion section of this article. The detailed mapping and meeting report are available from the WHO Department of Maternal, Newborn, Child and Adolescent Health – mncah@who.int.

## Results

**Figure 3:**
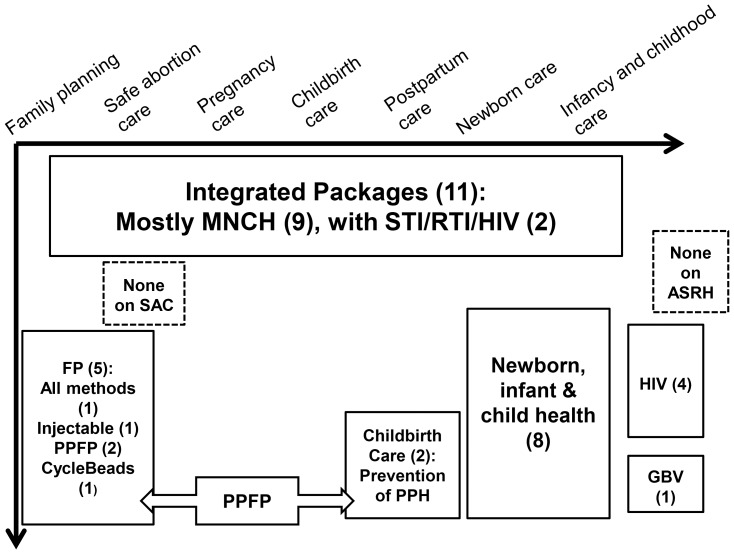
Distribution of identified training resource packages. By breadth (x axis) and depth (y axis) of coverage along the continuum of sexual and reproductive health and maternal, newborn, child, and adolescent health continuum (n = number of packages). Acronyms: ASRH: adolescent sexual and reproductive health, FP: family planning, GBV: gender-based violence, MNCH: maternal, newborn and child health, NB: newborn, PPFP: postpartum family planning, PPH: postpartum haemorrhage, RTI: reproductive tract infection, SAC: safe abortion care, STI: sexually transmitted infection.

We contacted 86 primary key informants and 33 secondary key informants, of which 79 (66%) answered. We identified 52 training resource packages of which 31 were considered relevant. Based on the health area, the materials were divided into integrated and focused packages. Integrated packages were defined as materials dealing with two of more health areas along the SR/MNCAH continuum, while focused packages mainly dealt with a single health area. Figure 3 shows the distribution of the training resource packages by breadth and depth of coverage of the SR/MNCAH continuum. Table 1 provides the list of training resource packages on SR/MNCAH for CHWs that were included in the mapping.^c^

**Table 1:** Training resource packages on sexual and reproductive health and maternal, newborn, child, and adolescent health.

INTEGRATED PACKAGES
• Community Health Worker Trainer’s Manual. Millennium Villages Project, 2011/2013 Update.
• Training manual for volunteers on maternal and newborn health care. Society for Family Health Revised edition, 2012.
• Timed and Targeted Counselling. World Vision International, 2010.
• Guide for Training Community Health Workers/Volunteers to Provide Maternal and Newborn Health Messages. USAID, POPPHI, BASICS, 2009.
• Training manual for cluster representatives and health volunteers, Module 1 and Module 3. WHO, 2009
• Community-Based Health Planning and Services (CHPS), Community Health Volunteers Training Manual. The Population Council, Ministry of Health/Ghana Health Service, 2009.
• Linking Communities with the Health System: the Kenya Essential Package for Health at Level 1. MOH Kenya, 2007.
• CHW Certificate Program – Facilitator’s Guide (selected modules). National Department of Health, Papua New Guinea, 2006.
• Reproductive Health Manual for Trainers of CHWs. CEDPA (Centre for Development and Population Activities), India, 2003.
• Health Surveillance Assistants Training Curriculum, MoH Malawi, 2006.
• Health Extension Worker Program of Training Packages Ethiopia. Ministry of Health Federal Democratic Republic of Ethiopia, The Earth Institute at Columbia University Centre for National Health Development in Ethiopia, 2003/2004.
**FOCUSED PACKAGES**
**Family planning**
• Population, Health and Environment (PHE) Community-based Distribution and Peer Education System: A Guide for Training PHE Community-based Distributors. Balanced Project (Coastal Resources Center, PATH Foundation Philippines, Conservation International), 2012.
• Training for community-based delivery of injectable contraceptives. FHI360, Child Fund Zambia, 2009 (2011 update).
• Postpartum Family Planning for Community Health Workers. Jhpiego, ACCESS-FP/USAID, 2010.
• Community Health Worker Postpartum Family Planning - Training Package for CHW Trainers. USAID, 2008.
• Offering CycleBeads: a Toolkit for Training Community Health Workers. Institute for Reproductive Health, Georgetown University, 2008.
**Childbirth care**
• Prevention of Postpartum Haemorrhage at Home Birth – A Program Implementation Guide. Access Program/Jhpiego. 2009.
• Prevention of postpartum haemorrhage at home birth – Facilitator’s guide and reference manual. MOH, Population Nepal, 2005.
**Newborn, infancy, childhood care**
• Caring for the newborn at home, a training course for community health workers. WHO, UNICEF, 2012.
• Care for child development: improving the care of young children. WHO, UNICEF, 2012.
• The Community Infant and Young Child Feeding Counselling Package: Facilitator Guide. UNICEF, URC/CHS, 2012.
• Caring for the sick child in the community. WHO, UNICEF, 2011.
• Manual de agente comunitario de salud: Atención integrada a las enfermedades prevalentes de la infancia (AIEPI) [IMCI]. PAHO, 2010.
• Community IMCI. MOH Cambodia, 2008.
• Infant young child feeding counselling: An integrated course. WHO, UNICEF, 2006.
• How to Train Community Health Workers in Home-Based Newborn Care. The SEARCH Team, 2006.
**HIV**
• Guide for Training Community Health Workers in WASH-HIV Integration. C-CHANGE (FHI360, Kenya MoPH and Sanitation), 2011.
• Accompagnateur Training Guide. Partners in Health, 2008.
• HIV Prevention Treatment Care and Support- A Training Package for Community Volunteers. WHO, IFRC, Safaids, 2006.
• Community Home-Based Care for People and Communities Affected by HIV: Training Course and Handbook for Community Health Workers. Pathfinder International, 2006.
**Gender-based violence**
• Rethinking Domestic Violence: A Training Process for Community Activists. Raising Voices, 2004.

These were identified from October to December 2012 (n = 31) and are classified by ‘integrated’, ‘focused’ (family planning, childbirth care, newborn, infancy and childhood care, HIV, gender-based violence), and date of publication, starting with the most recent.

### Coverage of the SR/MNCAH Continuum

#### Integrated packages

Integrated training resource packages were identified; 11 in total. They were all produced by partners, seven of which were developed at country level [30]–[40]. They mostly address the continuum of care of maternal, newborn and child health, with some including other components, such as sexually transmitted or reproductive tract infections, and HIV. Interventions in maternal health included the following components: recognition of pregnancy danger signs and referral; promotion of use of skilled attendant during pregnancy, birth and after birth; and promotion of adequate nutrition, iron and folate supplements, tetanus toxoid immunization, deworming, malaria prevention and intermittent preventive treatment, and post-partum family planning. Interventions for newborn care included, among others, post natal care visits in the first week after birth and the promotion and support for key newborn care practices including postnatal check-ups, exclusive breastfeeding, recognition of danger signs and timely care-seeking, immunization, and infection prevention (e.g. hand-washing with soap, cord care). As for child health interventions, they often covered the following topics: identification and referral of children with signs of severe illness including fever, diarrhea, and malnutrition; promotion of immunization and proper nutrition for the child’s age. At the global level, the UN H4+ agencies do not have an integrated package, and have concentrated on producing focused packages.

#### Focused packages

Family planning. Some of the integrated packages covered the promotion of family planning for birth spacing and healthy timing of pregnancy. Partners designed five family planning training resource packages on very focused topics, such as injectable contraceptives [41], postpartum family planning [42], [43], CycleBeads [44], or linking family planning and environmental sustainability [45].

Maternal health care. Promotion of use of skilled care during pregnancy care, childbirth and after birth was the main intervention addressed in the integrated packages. Partners also produced two focused training packages addressing postpartum haemorrhage prevention [46], [47].

Newborn, infancy, childhood care. UN H4+ agencies developed six training packages on newborn, infant and childhood care [48]–[50], while partners produced two [51], [52]. This is the area in CHW training where WHO and UNICEF have invested the most; WHO and UNICEF are in the process of finalizing an additional training package for community health workers to counsel caregivers on healthy growth and development, focusing on playing and communicating with the child.

HIV. UNAIDS and collaborating institutions developed a training package on HIV prevention, treatment, care and support for community volunteers (and not specifically targeting CHWs) [53], while partners developed three [54]–[56].

*GBV*. Partners produced one training package on the prevention of domestic violence through a process of change in the community, although it targets community activists rather than CHWs [57].

Other. Other areas were often included, particularly in country-based training packages, reflecting local public health priorities and important skills necessary for CHWs to perform their SR/MNCAH tasks, including interpersonal skills (across most curricula), record keeping (strong emphasis in many curricula), community mobilization (several curricula), integration of water sanitation and hygiene (WASH) with HIV in Kenya and Ethiopia [36], [40], treating minor ailments in the community in India (to increase acceptability of CHWs by the community), CHW self-care and wellbeing [56], first aid, waste disposal, food packages and others [40].

Summary. Both integrated and focused training packages often did not address all SR/MNCAH interventions recommended at community or home level in the UN H4+ packages of interventions (see Box 1) [25]. The search did not yield training resource packages for CHWs focusing on the specific areas of promotion of safe abortion-related services, GBV-related interventions, and adolescent SRH, although the promotion of safe abortion-related services (to the full extent of the law) and GBV-related interventions are part of WHO guidance [25]. As previously mentioned, the package dealing with GBV [57] does not specifically target CHWs but community activists.

### Training Considerations

Among the parameters used to review the different training resource packages, assessment of competencies and training follow-up and supervision showed some noticeable gaps:

#### Assessment of competencies

Competency is the successful demonstration of essential knowledge, skills, attitudes and professional behaviour on a specific task, action or function in the work setting [58]. While a majority of curricula are described as competency-based, assessment of the CHW trainees’ competencies is only applied in half (n = 16) of the identified training packages. The packages that do not carry out competency-based assessment usually apply pre- and post-testing of knowledge, which does not necessarily reflect the trainee’s competency [59].

#### Training methodology, follow-up and supervision

Most curricula indicate that they use adult learning principles in their training methodology. This is operationalized by using participatory, interactive and iterative methods. Many training courses concentrate on demonstration and practice with job aids, first in the classroom, then in households. Some materials cater to CHWs with no or very low literacy by using illustrated materials and focusing on practice with job aids and verification of memory [33]. However, capacity development is a continuous process of strengthening knowledge and skills and requires follow-up and supportive supervision of trainees, among other conditions, well beyond the actual training session [60]. This is not mentioned in more than a third (n = 12) of the training resource packages. Among the packages that mention its importance (n = 21), two thirds (n = 14) provide detailed recommendations on carrying out follow up and supportive supervision activities. Nevertheless, much of the supervision elements in these materials is focused more on the collection and analysis of data, and less on quality of care assurance, performance evaluation and feedback, and integration of existing community structures to oversee the activities of CHWs. A few packages have produced a more comprehensive CHW supervision manual [30], [32], [50].

#### Training course assessment

Information on whether the training course was evaluated was difficult to obtain, as it was not mentioned in the training resource packages, and follow up with the authoring agencies was challenging given the relatively short mapping time period. Further follow up is required. Information on training course assessment was obtained for slightly more than half (n = 18) of the training packages and indicate a wide range of breadth and depth in assessment. Some packages are currently undergoing evaluation [32], while others did not have the resources to undertake an evaluation process [49], although adaptation and implementation took place in several countries (personal communication). A few were thoroughly evaluated, such as the ACCESS Program on postpartum family planning package [46], which shows impact 12 to 18 months afterwards with increased postpartum family planning contraceptive prevalence rate, as well as increased use of antenatal care and skilled attendant at birth, however documentation was not available (personal communication).

#### Selection criteria of CHWs

The majority of training resource packages recommends the enrolment of trainees who have at least a basic degree of literacy equivalent to a 6^th^ grade primary school level. Higher literacy was regarded as particularly important if CHWs are to undertake more complex tasks, such as the distribution of Misoprostol in the context of postpartum haemorrhage prevention [47], or home-based clinical interventions for the sick child [22]. But as mentioned, several materials also cater to non-literate trainees, and usually provide illustrated materials, including reporting charts [33], and a strong focus on practice with job aids and verification of memory. Ideally, the trainee must come from the local community, and be “respected, creative, hardworking and responsible” [36], lead by example, and be willing to work for a period of at least five years. Preference is given to women in certain programmes.

#### Duration of the training course

Duration can span from two to three hours for CycleBeads [44] to a year in Ethiopia’s extension worker programme [40], or even two years in Papua New Guinea’s CHW programme [37]. Note that the Ethiopia and Papua New Guinea curricula are intended for more advanced community-based service providers with service delivery skills equivalent to those of an auxiliary nurse. In general, the majority of training courses last between three to five days, for either focused or integrated packages. Some curricula are delivered in several phases and include month-long practice intervals between classroom sessions [32], [36].

#### Integration into national health system

It was difficult to obtain information with regard to the integration of the training packages and of the trained CHWs into national health systems as the information is not readily available in the training resource packages and was challenging to obtain through follow up with authoring agencies. More than half of the packages identified (55%) were global or regional generic materials; 45% were national materials which may give some indication of lack of integration or reflect the limitations of the search. Only one package provided a separate accompanying resource to guide country level integration [32].

#### Cost of training

The cost was either unknown for a majority of packages or when known, varied depending on the setting where the training course took place. In other words, they did not provide a standard estimated cost.

## Discussion

The research yielded a fair number of existing training resource materials that were useful to inform a qualitative analysis of the situation regarding training CHWs in SR/MNCAH. The identification of these materials faced several constraints, including a relatively short timeframe to follow up with key informants and the inclusion of only materials made publicly available, although organizations might have valuable training packages that are not in the public domain. The search also concentrated on the SRH aspect of adolescent health and did not search for other terminology, such as peer-to-peer educators, which may be particular to adolescent programmes. In spite of the limitations, the study manages to provide an illustration of existing training materials and an analysis of gaps and opportunities for the field:

### Technical Gaps from Existing Tools

Overall there is a large number of training resource packages for CHWs on different components of SR/MNCAH. Many are overlapping but some also integrate other topics that are relevant to a particular context, such as linking family planning with environmental sustainability in the Philippines or WASH with HIV in Kenya and Ethiopia. The majority of these packages were developed by the UN H4+ and partners over the past decade, many before recommendations were issued on evidence-based interventions for CHWs. Based on the most recent WHO/UN H4+ guidance [25], several key interventions were found to be missing, for example, informing about the availability of pregnancy detection and safe abortion services, and education on the consequences of unsafe abortion; recognition of postpartum blues/depression and referral; promotion and support for birth registration; or promotion and support for child stimulation and play. These could be addressed in the revision of existing training packages or the crafting of new ones, according to country needs. Importantly, no integrated or focused training resource package addresses all the WHO/UN H4+ recommended core interventions to be delivered by CHWs. Attention needs to be given to regularly updating and aligning the technical contents of the packages with global guidance, especially in rapidly evolving areas, such as HIV [61]. In addition, some of the topics covered by the training packages may not have a sufficient evidence base to be recommended for scale up and need to be considered only in the context of rigorous research/documented supervision (e.g. insertion of contraceptive implants by CHWs), or with targeted monitoring and evaluation (e.g. initiation and maintenance of injectable contraceptives by CHWs) [27].

No published training resource package for CHWs dedicated to adolescent SRH, safe abortion, and GBV were identified, although community-based interventions for the promotion of safe abortion-related services (to the full extent of the law) and elements of GBV activities are part of the WHO/UN H4+ recommendations. These topics are sensitive, and perhaps even more so where CHWs come from the same local communities as the persons concerned. This raises the question of whether CHWs can effectively include sensitive and potentially stigmatising topics, such as adolescent SRH and GBV, into their household visits. Due to the public health importance of these topics, another way to support CHWs in these areas of work would be to ensure that they mobilize the communities and raise their awareness, instead of dealing with these sensitive topics at the household level. However, in case a woman spontaneously discloses violence, CHWs should at least have the skills to listen carefully and empathetically, be non-judgmental and supportive, and help her access information about resources and services if these are available [62]. Lessons learnt could be drawn from the HIV field where stigma and discrimination are also impediment to prevention, treatment, care and support interventions [63], [64]. This calls for further implementation research.

### Impact of Training

The relatively short time frame limited the follow up with key informants, authors or authoring agencies. Further effort would be required to determine the overall impact of training resource packages. In this regard, evaluation of training programmes for CHWs is an area with limited published research and many agencies did not have evaluation reports readily available. There is a need to define agreed indicators to help programmes monitor and evaluate their trainings in the medium to longer term, including impact evaluation. But this is an area that is not exclusive to CHW training as it relates to training health workers in general [20].

### Health System and Programmatic Considerations

Beyond the clinical contents, other gaps that have emerged from the review of the training resource packages have to be addressed. These gaps are reflected in recently published literature on CHWs [18] and include the lack of high standard guidelines for the supervision and quality assurance of CHW activities, the weak integration of CHWs into the community health system, and cross-sectoral integration of CHWs as part of the overall health system strengthening. These gaps reflect the critical importance of embedding CHW training initiatives into wider programmatic and health system strengthening considerations, including ensuring an enabling policy environment, establishing clear linkages to other levels of care and health professionals, and ensuring that they are equipped with appropriate supplies and materials, supported, remunerated, motivated, and supervised.

Important questions still need to be answered to inform programmes and policies. Some questions are specific to SR/MNCAH, such as the role of CHWs in maternal and perinatal death surveillance and response [65]. Other questions are not specific to SR/MNCAH and include CHW programme cost, the use of mobile technologies in CHW interventions, most equitable and cost-effective ways to reach populations living in remote areas, or the optimal number and distribution of CHWs in different populations, including in hard-to-reach settings.

### Moving Forward

Taking into account the wealth of available training packages, identified gaps, programmatic and health system considerations, as well as the diverse needs of countries and programmes, three different but complementary deliverables could be considered for development at the global level to support countries and partners implement an effective and harmonized approach to training CHWs on SR/MNCAH. Any material would need to be adapted to the context.

#### 1. Orientation material

This material would support district health management teams and programme managers to consider key programmatic and health system considerations to not only implement the core training resource package and additional modules described above, but also effectively put in place and improve CHW programmes on SR/MNCAH as part of a health system strengthening approach. The contents of this orientation material would cover guiding principles in implementing CHW trainings and programmes (see Box 1).

#### 2. Core training resource package

This material would target facilitators and CHW trainees and would therefore be packaged into a facilitator’s guide and participant’s manual. It would be intended to streamline existing CHW work rather than adding burden. The contents would cover: i) a technical orientation on core SR/MNCAH interventions for CHWs (mainly community engagement, health promotion, information, counseling, education, support and identification of health conditions and referral), using as references recent WHO technical guidance; ii) core competencies that are not specific to the SR/MNCAH domain, but are critical for CHWs to effectively carry out their functions in general, and that include for instance: knowing your community, interpersonal communication, behavior change frameworks, privacy and confidentiality, organizing and planning, record keeping, referrals, team work, and others. Among the 33 training resource packages reviewed, several quality materials can be considered as a reference for developing this core training resource package.

#### 3. Additional technical modules on specific SR/MNCAH areas

Building on the basic foundation provided by the core training resource package, additional technical modules that require higher-level skill training could be included to broaden the range of SR/MNCAH interventions provided by CHWs, such as the provision of curative care, running rapid tests and managing the results, or the distribution of medicines and supplies. These additional technical modules should complement and not supplant the essential interventions and core competencies defined in the core package, and therefore, their scalability should be carefully informed by wider programmatic and feasibility considerations. They should be designed in such a way to allow seamless integration with the core training resource package, including in competencies, supervision and quality assurance systems.

## Conclusions

We provide an illustrative and qualitative synthesis of the current landscape of SR/MNCAH training resource packages for CHWs to inform reflection on ways to establish a harmonized approach to strengthening capacity. Rigorous documentation and evaluation of CHW training programmes will provide further evidence to inform capacity strengthening efforts. The wealth of training resource packages indicate not only the growing momentum to reposition the role of CHWs in national health systems so that they can contribute to accelerated progress on the health MDGs, but also the large investment made by many partners. Recent advances in rapid diagnostic tests that can be performed at household level and mobile technology for health, combined with new political will and resources, offer a unique opportunity for scaling up CHW programmes that can have a positive impact on maternal, newborn and child mortality. This momentum also offers a window of opportunity for strategic partnership among key stakeholders to mount a synergistic response to address the identified gaps and ensure a harmonized, evidence-based and effective approach to developing the capacity of CHWs in SR/MNCAH. CHWs can be an integral part of strong health systems that equitably deliver key information and services to the whole community.
